# Associations Between Fetal Growth Trajectories and the Development of Myopia by 20 Years of Age

**DOI:** 10.1167/iovs.61.14.26

**Published:** 2020-12-23

**Authors:** Kathleen I. C. Dyer, Paul G. Sanfilippo, Scott W. White, Jeremy A. Guggenheim, Chris J. Hammond, John P. Newnham, David A. Mackey, Seyhan Yazar

**Affiliations:** 1 Centre for Ophthalmology and Visual Science, Lions Eye Institute, University of Western Australia, Perth, Western Australia, Australia; 2 School of Medicine, Faculty of Health and Medical Sciences, University of Western Australia, Perth, Western Australia, Australia; 3 Centre for Eye Research Australia, Department of Ophthalmology, University of Melbourne, Royal Victorian Eye and Ear Hospital, Melbourne, Victoria, Australia; 4 Division of Obstetrics and Gynaecology, Faculty of Health and Medical Sciences, University of Western Australia, Perth, Western Australia, Australia; 5 Maternal Fetal Medicine Service, King Edward Memorial Hospital, Perth, Western Australia, Australia; 6 School of Optometry and Vision Science, Cardiff University, Cardiff, South Glamorgan, United Kingdom; 7 Department of Twin Research and Genetic Epidemiology, King's College London, London, United Kingdom; 8 Garvan Institute of Medical Research, Sydney, New South Wales, Australia

**Keywords:** myopia, ocular biometry, fetal growth, trajectory modeling, birth cohort

## Abstract

**Purpose:**

To evaluate the contribution of genetic and early life environmental factors, as reflected by fetal anthropometric growth trajectories, toward the development of myopia during childhood and adolescence.

**Methods:**

This analysis included 498 singleton Caucasian participants from the Raine Study, a pregnancy cohort study based in Western Australia. Serial fetal biometric measurements of these participants were collected via ultrasound scans performed at 18, 24, 28, 34, and 38 weeks’ gestation. At a 20-year follow-up, the participants underwent a comprehensive ophthalmic examination, including cycloplegic autorefraction and ocular biometry measurements. Using a group-based trajectory modeling approach, we identified groups of participants with similar growth trajectories based on measurements of fetal head circumference (HC), abdominal circumference, femur length (FL), and estimated fetal weight (EFW). Differences between trajectory groups with respect to prevalence of myopia, axial length (AL), and corneal radius of curvature measured at the 20-year follow-up were evaluated via logistic regression and analysis of variance.

**Results:**

Prevalence of myopia was highest among participants with consistently short or consistently long FLs (*P* = 0.04). There was also a trend toward increased prevalence with larger HC in late gestation, although not at a statistically significant level. Trajectory groups reflecting faster HC, FL, or EFW growth correlated with significantly flatter corneas (*P* = 0.03, *P* = 0.04, and *P* = 0.01, respectively) and a general, but not statistically significant, increase in AL.

**Conclusions:**

Environmental or genetic factors influencing intrauterine skeletal growth may concurrently affect ocular development, with effects persisting into adulthood.

Myopia arises when the eye's refractive power is too strong for its axial length, causing images to be focused in front of the retina. This may be due to an elongated axial length (AL), a short corneal radius of curvature (CR), or a combination of these features.^1^ Myopic error typically presents during school age and progresses as the eye continues to grow throughout childhood and adolescence.^1^ Therefore, risk factors for myopia are likely linked to the development of the cornea, lens, and sclera, which must be closely coordinated to accurately correlate AL with refractive power as the eye increases in size from the 10th week of gestation until approximately 11 to 12 years of age.^2^^–^^5^ Conditions that disrupt this coordinated process during either fetal life or childhood may result in a refractive error.

In recent decades, the worldwide prevalence of myopia has risen at an alarming rate, and current estimates suggest that 50% of the global population will be myopic by the year 2050.^6^ There is currently no cure for myopia, and this condition remains a major risk factor for potentially blinding diseases, including retinal detachment, glaucoma, macular degeneration, and cataract formation, particularly with high myopia (defined as ≤–6 diopters [D]).^6^^,^^7^ Therefore, a clear understanding of the risk factors contributing to the development of myopia, including those present during gestation, is urgently needed to determine optimal preventative strategies.

Factors influencing the processes involved in prenatal ocular growth may be reflected in intrauterine skeletal growth. Indeed, neonatal anthropometry has previously been shown to correlate with ocular morphology in both childhood and adulthood in a number of studies; however, the evidence for a corresponding difference in refractive error is conflicting.^8^^–^^16^ Moreover, many of these studies have used cross-sectional data only, often with a wide variation in age at follow-up. Recently, findings from a Dutch prospective birth cohort study demonstrated an association between fetal growth trajectories based on estimated fetal weight (EFW) and both AL and CR at 6 years of age.^17^ However, it is unknown whether this relationship persists after the attenuation of eye growth and whether there is a corresponding correlation between fetal growth trajectory and the risk of myopic error measured in the fully grown eye. Furthermore, there are as yet no studies that have examined individual associations of AL, CR, or myopia with gestational head circumference (HC), abdominal circumference (AC), and femur length (FL) growth, each of which reflects different aspects of fetal development.^18^

The aim of this study was to evaluate in detail the contribution of genetic and early life environmental factors toward the development of myopia during childhood and adolescence, specifically by analyzing the relationships between fetal growth trajectories based on longitudinal measurements of HC, AC, FL, and EFW, as reflective of such factors, and the risk of developing myopia by around 20 years of age, in addition to concurrently measured ocular biometry parameters of AL and CR.

## Methods

### Study Design

The Raine Study is a prospective multigenerational epidemiologic study based in Perth, Western Australia, that aims to evaluate the contribution of early life influences toward health outcomes in child and adult life. The original design incorporated a randomized controlled trial to investigate the effects of serial fetal ultrasound scans during pregnancy on birth outcomes.^19^ The study enrolled 2900 pregnant women at King Edward Memorial Hospital between 1989 and 1991. Mothers were recruited at 16 to 18 weeks’ gestation from the King Edward Memorial Hospital antenatal clinic and nearby private clinics. Participating mothers (Gen1 participants) were then randomized at a ratio of 1:1 to either “intensive care” involving ultrasound assessment, including fetal biometry measurements and Doppler assessment of umbilical artery flow at 18, 24, 28, 34, and 38 weeks’ gestation, or “regular care” comprising ultrasound imaging at 18 weeks’ gestation and only as clinically indicated thereafter.^19^ There were 2868 live births in the original cohort, and since birth, these offspring (Gen2 participants) have been assessed for a wide variety of health-related outcomes at 1, 2, 3, 5, 8, 10, 14, 17, 20, 22, 27, and 28 years of age as a prospective cohort study.^20^ A comprehensive ophthalmic examination was conducted for the first time at the Gen2-20 year follow-up, to which all active participants were invited.

The Raine Study is registered in the Australian New Zealand Clinical Trials Registry.^21^ Written informed consent was obtained by all Gen1 participants upon enrollment. The protocol for ultrasound measurements was approved by the ethics committees at King Edward Memorial Hospital, Princess Margaret Hospital, and the University of Western Australia. All participants at the Gen2-20 year follow-up provided their own informed consent, and the Human Research Ethics Committee at the University of Western Australia approved the protocol for ophthalmic data collection. The protocol for all data collection during gestation and at the Gen2-20 year follow-up adhered to the Declaration of Helsinki.

### Participants

**Figure 1.**
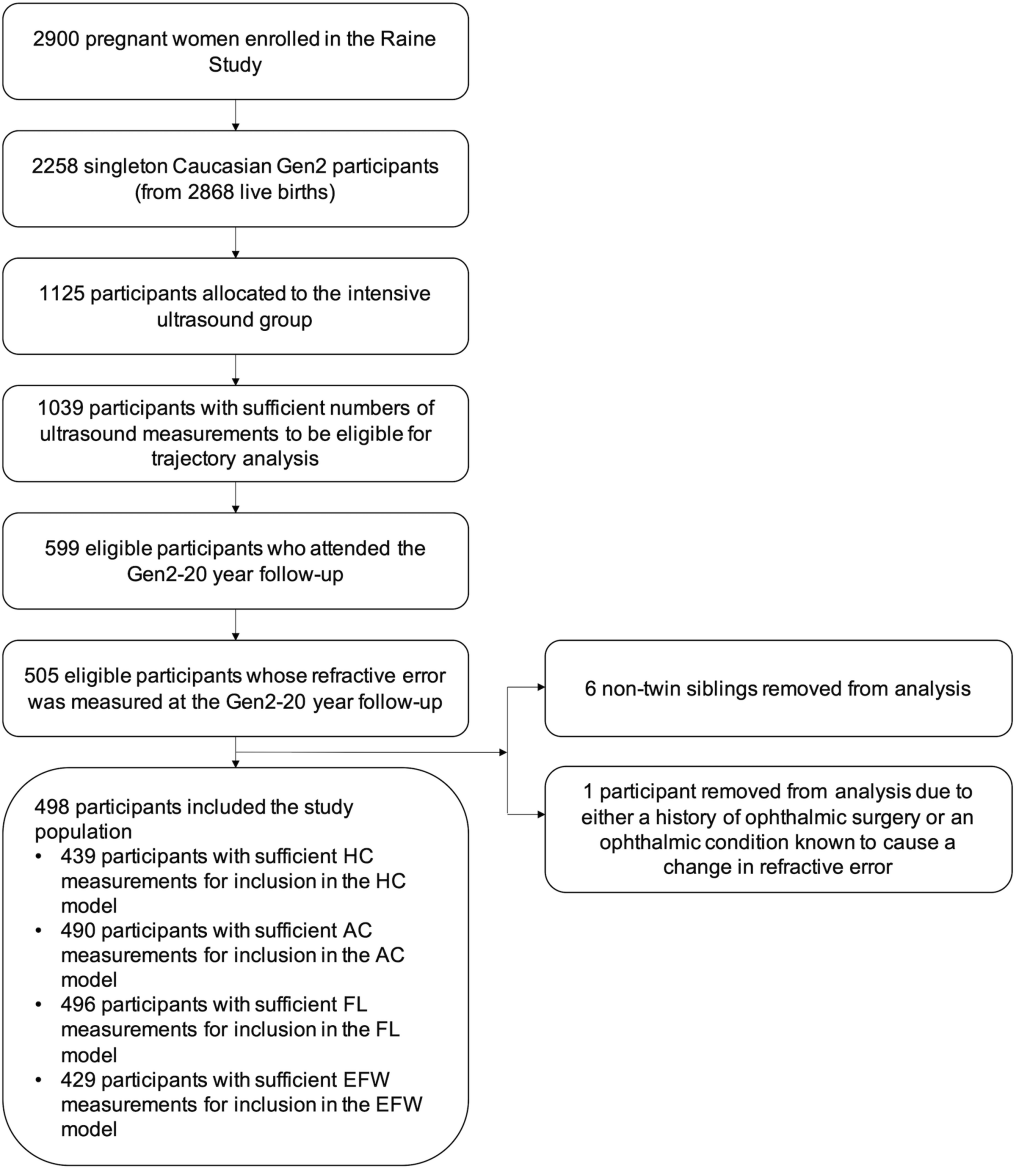
Study population flowchart. “Sufficient numbers of ultrasound measurements” was defined as least one fetal biometric parameter measured around at least four of the nominal time points during gestation. “Sufficient measurements” of a specific fetal growth parameter for inclusion in the corresponding trajectory model was defined as measurements of that fetal growth parameter around at least four of the nominal time points during gestation. The one participant removed on the basis of ophthalmic history was specifically excluded due to a history of keratoconus.

Our analysis included 498 singleton Caucasian Raine Study Gen2 participants for whom ultrasound measurements of at least one fetal biometric parameter were recorded around at least four of the five nominal time points (18, 24, 28, 34, and 38 weeks’ gestation), and refractive error measurements were recorded at the Gen2-20 year follow-up (see Fig. 1). To reduce the possibility of introducing a selection bias, only participants from the “intensive care” ultrasound group were included, since there is a higher likelihood that mothers in the “regular care” ultrasound group with four or more ultrasound measurements had medical or gestational conditions requiring them to undergo additional scans. The study population was further restricted to Caucasian participants as only approximately 15% of participants with sufficient ultrasound and ophthalmic measurements for this study had parents of non-Caucasian descent, rendering the statistical power to examine the specific confounding effects of ethnicity in our analysis insufficient. In addition, participants from multiple pregnancies (i.e., twins and triplets) were excluded due to the higher potential for pregnancy complications, including those specific to multiple pregnancies, that may affect fetal growth^22^ and that are not examined in this analysis. Where multiple nontwin siblings were Gen2 participants, only one randomly chosen sibling was included in the analysis to reduce bias due to genetic similarity. Participants with any history of ophthalmic surgery or any ophthalmic condition known to cause a change in refractive error, such as keratoconus, corneal abrasions or ulcers, cataracts, or nystagmus, were also excluded.

### Data Collection

Baseline maternal characteristics were collected via questionnaires completed by Gen1 participants at 16 and 34 weeks’ gestation. Pregnancy outcomes, including medical conditions during gestation and birth parameters, were recorded by research midwives from a review of medical records. Serial measurements of fetal HC, AC, and FL and umbilical artery Doppler flow velocity waveforms were obtained via ultrasound imaging by qualified sonographers with one of two General Electric 3600 machines (Milwaukee, WI, USA), using standard anatomical landmarks.^19^ EFW was calculated using a Hadlock formula based on HC, AC, and FL measurements.^23^

Techniques for ophthalmic data collection at the Gen2-20 year follow-up have previously been described in detail by Yazar et al.^24^ In brief, AL and CR were measured using an IOLMaster V.5 (Carl Zeiss Meditec AG, Jena, Germany). Autorefraction was performed with a Nidek ARK-510A (NIDEK Co. Ltd., Gamagori, Japan) both before and after the administration of cycloplegic eye drops (one drop tropicamide 1%, one drop phenylephrine 10%). For this study, all analyses of the ophthalmic outcomes were performed using measurements of the right eye only. Refractive error was calculated as the mean spherical equivalent, that is, the sum of the spherical error and half the cylindrical error, based on the postcycloplegic measurements. Myopia was defined as a mean spherical equivalent of ≤−0.5 D. A camera system developed by Ooi et al.^25^^,^^26^ was used to perform measurements of the total area of conjunctival ultraviolet autofluorescence (CUVAF) across both eyes, which is an objective marker of an individual's sun exposure.^27^ Data relating to education level and parental myopia were collected via questionnaires. In this analysis, education level was defined based on completion of year 12 in Australia or equivalent (International Standard Classification of Education Level 3).^28^

### Statistical Analyses

We constructed trajectory models for each fetal growth parameter as follows, using data from all participants in our study population with ultrasound measurements of the parameter recorded around at least four of the five nominal time points previously listed. First, a standard deviation score (SDS) associated with each fetal biometry measurement was calculated. This was performed by constructing a linear mixed-effects model to examine the relationship between the fetal growth parameter and gestational age (linear and quadratic terms). Maternal height, maternal age, and fetal sex were included as fixed effects in the model, in view of the known influences of these three baseline characteristics on fetal growth.^18^ Random effects for gestational age (intercept and slope) were also fitted to account for individual variation about the mean. The fetal growth parameter being examined was transformed prior to model construction to satisfy the model assumption of constant variance in the residuals (square root transformations for HC and FL and logarithmic transformations for AC and EFW). Continuous covariates (gestational age, maternal height, and maternal age) were centered to ensure model convergence. For each fetal growth parameter, the associations with all covariates in the corresponding linear mixed-effects model were significant (*P* < 0.05), except those between HC and maternal age (*P* = 0.06), AC and fetal sex (*P* = 0.15), AC and maternal height (*P* = 0.16), and EFW and fetal sex (*P* = 0.41); nonetheless, all covariates were retained in all four models for consistency. The marginal residuals from each model (i.e., the residuals calculated from predicted values based on the fixed effects) were then standardized to create SDSs corresponding to each fetal biometry measurement.

We used a group-based trajectory modeling (GBTM) approach to compute trajectory models based on the SDSs. Developed by Nagin,^29^ GBTM is a method designed to identify clusters (or “groups”) of individuals with similar developmental trajectories, that is, similar patterns of evolution over time with respect to a longitudinally measured variable. Each individual is then assigned to a group according to probability of membership. Unlike similar trajectory modeling techniques such as latent-curve modeling, GBTM allows clusters to naturally emerge from the data even when they are not necessarily identifiable on the basis of known individual traits or exposures.^29^ Although fetal growth trajectory may be influenced by feto-maternal characteristics that are known ex ante, these characteristics are not necessarily associated with clearly distinguishable trajectories, and as such, GBTM was considered the most appropriate technique for this study. For each fetal growth parameter, we determined the optimal trajectory model for analysis as follows, in accordance with procedures outlined by Nagin^29^ and Nagin and Odgers.^30^ We computed trajectory models of 1 to 10 SDS trajectory groups with either linear-shaped trajectory groups or quadratic-shaped trajectory groups. Models with different numbers of groups were compared in a stepwise manner, and we also compared each linear trajectory model to the quadratic trajectory model with the same number of groups. The optimal model for each fetal growth parameter was selected based on the interpretability of the grouping and the differences in the Bayesian information criterion (BIC) values between the models, with a higher BIC signifying a better model fit. We also required each model group to contain a minimum of 5% of the population included in the model and to have an average posterior probability of more than 70%.

Trajectory groups in the selected models were compared with respect to maternal and pregnancy characteristics, known risk factors for myopia, and the ophthalmic outcomes. The significance of any differences was evaluated using logistic regression for categorical variables and analysis of variance for continuous variables or Kruskal-Wallis tests for nonparametric continuous variables. The maternal and pregnancy characteristics examined included baseline maternal age, height, prepregnancy weight and parity, rate of gestational weight gain, fetal sex, and the gestational conditions or exposures of hypertension in pregnancy (including superimposed preeclampsia), diabetes (preexisting or gestational), gestational anemia, smoking during pregnancy, and abnormal Doppler flow. There were insufficient numbers of mothers with gestational diabetes or preeclampsia to evaluate the differences between trajectory groups with respect to these conditions alone. The known risk factors for myopia that were compared across trajectory groups were gestational age at birth, birth weight adjusted for gestational age, parental myopia (categorized as neither, one, or both parents), and total area of CUVAF and educational level recorded at the Gen2-20 year follow-up. Where any of parental myopia, total area of CUVAF, or educational level was found to be significantly correlated with group membership in a trajectory model, the analyses for associations between group membership in that model and the ophthalmic outcomes were adjusted for that specific risk factor for myopia. Adjustments were not made for gestational age at birth or birth weight, even if these correlated with group membership in a trajectory model, to avoid distorting the effects of these variables in the analysis due to the fact that these variables, like trajectory group membership, are also markers of fetal growth. The computation of group-based trajectory models was performed using the procedure Proc Traj within SAS statistical software, version 9.4 (SAS Institute, Inc., Cary, NC, USA).^31^ All other analyses were completed using R statistical software, version 1.1.456 (R Foundation for Statistical Computing, Vienna, Austria).

## Results

### Fetal Growth Trajectories: Descriptive Results

Of the 498 Gen2 participants included in this study, sufficient measurements for inclusion in the HC, AC, FL, and EFW trajectory models were recorded for 439, 490, 496, and 429 participants, respectively. For each of the four fetal growth parameters, there was successive improvement in the goodness of fit of the linear trajectory models with increasing numbers of groups according to the BIC, until the six-group HC model, eight-group AC model, five-group FL model, and eight-group EFW model were reached. Thereafter, the BIC decreased with increasing numbers of groups, suggesting poorer fit. However, to ensure a proportion of at least 5% of the population in each group, the five-group HC model, four-group AC model, five-group FL model, and six-group EFW model were selected for further analysis. Each of these models also demonstrated average posterior probabilities of more than 70% associated with all trajectory groups. Models with quadratic-shaped trajectory groups showed poorer model fit according to the BIC compared with the linear trajectory models and so were not retained.

**Figure 2.**
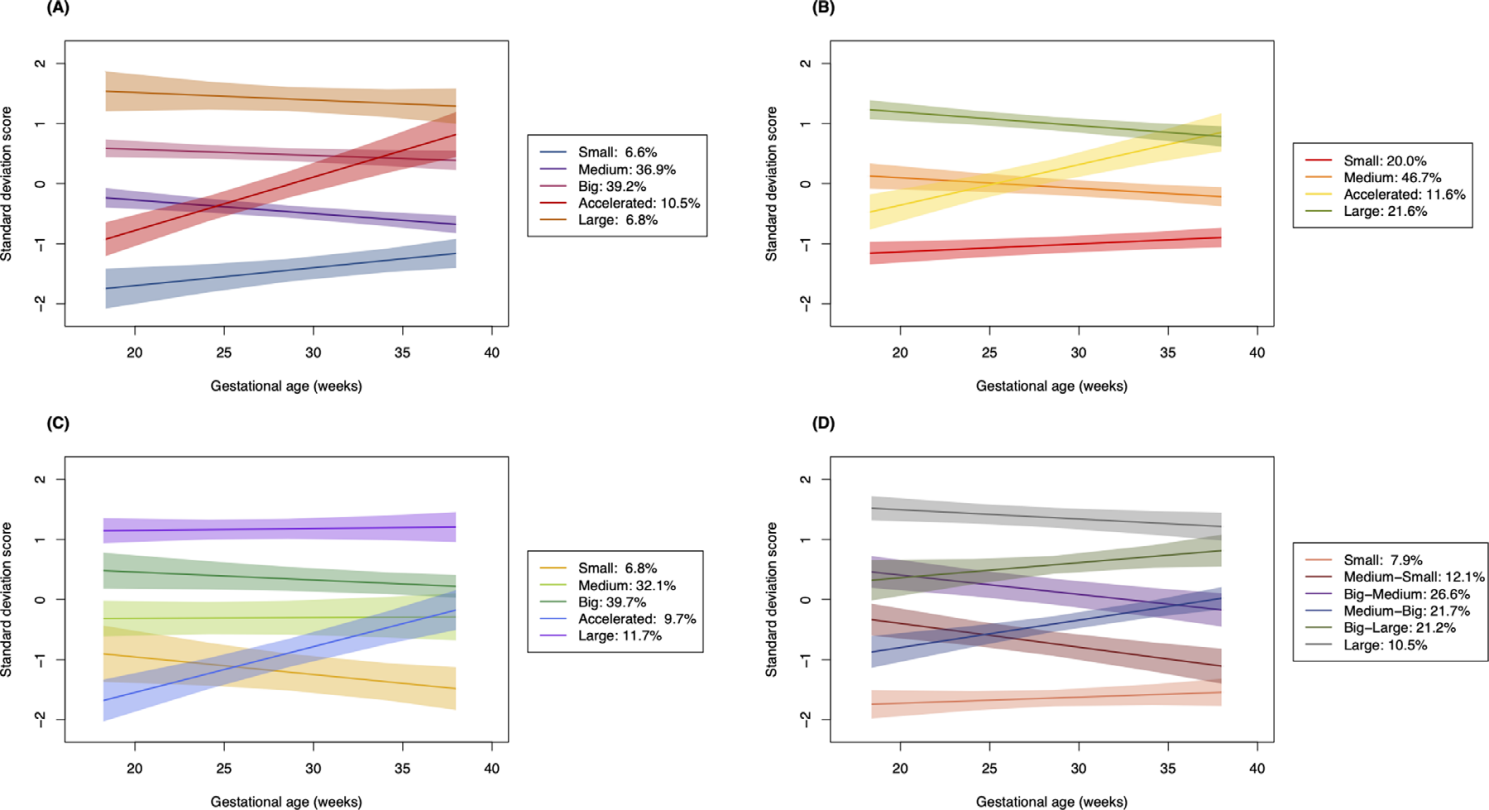
Plots of the trajectory groups (estimated mean trajectories with 95% confidence intervals). (**A**) The five-group head circumference trajectory model (*n* = 439). (**B**) The four-group abdominal circumference trajectory model (*n* = 490). (**C**) The five-group femur length trajectory model (*n* = 496). (**D**) The six-group estimated fetal weight trajectory model (*n* = 429).

Plots of the trajectory groups of the four selected models are displayed in Figure 2. The individual profiles of three randomly chosen subjects from each trajectory group in the HC, AC, FL, and EFW models are shown in Supplementary Figures S1, S2, S3 and S4, respectively, as an illustration of the in-group variabilities in trajectories. The selected HC and FL models showed four relatively stable trajectory groups reflecting participants who were consistently “small”, “medium”, “big”, and “large” throughout gestation with respect to the specific growth parameter. There were three stable trajectory groups in the AC model (“small”, “medium”, and “large”) and only two in the EFW model (“small” and “large”). All three of the HC, AC, and FL models also included an “accelerated” trajectory group. These groups reflected fetuses whose growth rate increased throughout gestation, relative to the general study population. The EFW model showed no clear stable medium-sized group: all four medium-sized groups showed moderate amounts of either deceleration (the “medium-small” and “big-medium” groups) or acceleration (the “medium-big” and “big-large” groups).

Supplementary Table S1 displays the maternal and pregnancy characteristics of participants in each model group. Distributions of maternal age, height, prepregnancy weight, rate of gestational weight gain, and parity and fetal sex were generally consistent across model groups, aside from in the FL model, which showed increased maternal prepregnancy weight and rate of gestational weight gain in the “accelerated” and “large” groups compared with the other trajectory groups. With regard to gestational medical conditions, the prevalence of hypertension in pregnancy was highest among individuals in the “accelerated” and “large” HC groups and the “small” FL group. Both diabetes (preexisting or gestational) and gestational anemia were most prevalent in either the “large” or “accelerated” group of the HC, AC, and EFW models, while abnormal Doppler flow occurred most often in the “small” or “medium” HC, AC, and EFW groups. Smoking during pregnancy was consistently associated with slower fetal growth, being most prevalent in the “small” or “medium” groups of every model.

The distributions of known risk factors for myopia are displayed in Supplementary Table S2. Gestational age at birth was lowest in the “large” HC group but otherwise did not vary substantially across trajectory groups in the other models. Birth weight adjusted for gestational age was considerably larger in groups representing faster growth with respect to all fetal growth parameters (*P* < 0.01 in all trajectory models). Total area of CUVAF and education level at 20 years of age were generally consistent across trajectory groups. The proportion of participants with either at least one parent with myopia was greatest in the “large” groups of all four models, and this difference reached statistical significance in the AC model (*P* < 0.01). Analyses for associations between AC trajectory group membership and the ophthalmic outcomes, reported below, were therefore performed both with and without adjustment for parental myopia.

### Relationship of Fetal Growth Trajectories to Ophthalmic Outcomes

**Figure 3.**
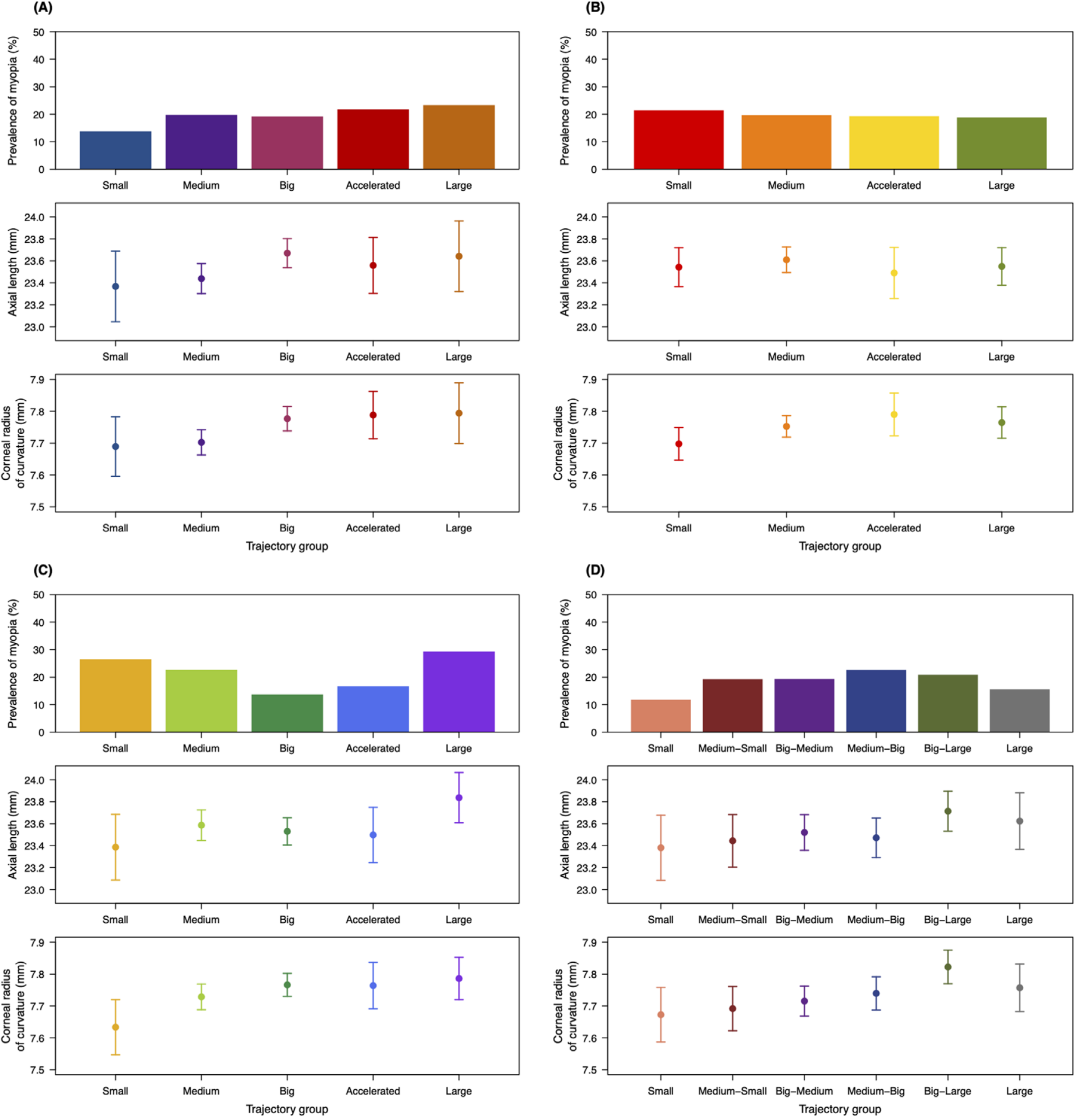
Ophthalmic characteristics of participants in the trajectory groups of each model. (**A**) Head circumference model. (**B**) Abdominal circumference model. (**C**) Femur length model. (**D**) Estimated fetal weight model. Each panel presents the prevalence of myopia (*top*), mean axial length (*middle*), and corneal radius of curvature (*bottom*) for each model group. Bars represent 95% confidence intervals calculated in a one-way analysis of variance. For myopia, *P* < 0.05 in the femur length model and *P* > 0.05 in all other models; for axial length, *P* > 0.05 in all models; and for corneal radius of curvature, *P* > 0.05 in the abdominal circumference model and *P* < 0.05 in all other models.

The ophthalmic characteristics of participants by trajectory model group are presented in Figure 3 and Supplementary Table S3. A total of 98 participants (19.7% of the study population) were myopic. The prevalence of myopia varied between trajectory groups and was associated with group membership in the FL model at a statistically significant level (*P* = 0.04). A U-shaped trend was shown in this model, in which the “small” and “large” groups had the highest rates of myopia (26.5% and 29.3%, respectively) while lower rates of myopia were found in the “medium”, “big”, and “accelerated” groups, which all contained participants with more moderate FLs in later gestation. In contrast, the HC model showed a trend of increasing prevalence of myopia with larger HC in late gestation, although this relationship did not reach a statistically significant level (*P* = 0.90). More specifically, the “small” trajectory group was associated with a markedly lower prevalence of myopia than the other groups (13.8%), while the trajectory group with the highest prevalence was the “large” group, followed by the “accelerated” group (23.3% and 21.7%, respectively). The prevalence of myopia was generally consistent across the AC trajectory model groups, with no significant correlation identified with or without adjustment for parental myopia (*P* = 0.97 and *P* = 0.77, respectively). The EFW model did not show a similar pattern to any of the other models with respect to myopia: the “small” and “large” groups had the lowest prevalence (11.8% and 15.6%, respectively) compared with the other four groups, which all had similar rates of myopia to each other. As EFW was calculated based on HC, AC, and FL, this pattern may reflect an offset between the relationships demonstrated in each of the three models described above.

The mean (standard deviation) AL and CR for the study population were 23.57 (0.89) mm and 7.75 (0.26) mm, respectively. There was a clear trend in the distributions of these ocular biometry parameters among the groups in each model. The HC model showed a general increase in mean AL and mean CR for groups with larger HC in late gestation (the “big”, “accelerated”, and “large” groups) compared with groups with smaller HC in late gestation (the “small” and “medium” groups). Likewise, larger fetal size in late gestation correlated with increasing AL and CR in the EFW model, in which the longest eyes and flattest corneas were seen in the “big-large” and “large” groups, while the “small” and “medium-small” groups showed the shortest mean AL and mean CR. Interestingly, although the FL model showed a similar trend with respect to CR, and the shortest and longest mean ALs were found in the “small” and “large” groups, respectively, of the other three groups, the “medium” group had both the longest mean AL and mean CR. This may account for the relatively high prevalence of myopia in this group compared with the two other groups containing participants with moderate-sized FLs in late gestation (“big” and “accelerated”). The only associations to reach statistical significance were those between CR and group membership in the HC, FL, and EFW models (*P* = 0.03, *P* = 0.04 and *P* = 0.01, respectively). The AC model also showed a shorter mean CR in the “small” group but no clear trend in the AL distributions between the model groups or statistically significant differences between the model groups with respect to either AL or CR (*P* = 0.79 and *P* = 0.13, respectively), including when adjusted for parental myopia (*P* = 0.71 and *P* = 0.37, respectively).

## Discussion

This analysis aimed to investigate possible associations between fetal growth trajectories and myopia, AL, and CR measured in a cohort of Caucasian adults aged around 20 years. As distinct from previous similar studies, we analyzed fetal growth trajectories based on measurements made during gestation of the individual growth parameters HC, AC, and FL, in addition to a summary growth parameter EFW, rather than EFW alone. The most notable finding was the existence of trends suggesting a close association between myopia and intrauterine skeletal development, particularly as reflected by FL growth trajectory. Specifically, the results showed a statistically significant U-shaped association between FL growth trajectory and prevalence of myopia as well as a trend toward an increased prevalence of myopia with larger HC in late gestation that did not reach statistical significance.

The observed trend toward increased prevalence of myopia with larger HC in late gestation, although not statistically significant, suggests that fetal HC development may play a small role in the development of refractive error. If so, this trend could be interpreted in terms of the effect of HC growth on ocular biometry. A 2019 analysis from within the Generation R Study,^17^ which is the other published investigation to have analyzed the association between fetal growth trajectory and ocular biometry in early childhood using fetal biometric measurements performed during gestation rather than postnatal surrogate markers, demonstrated an increase in AL and decrease in CR in 6-year-olds with increasing rate of fetal growth. In this case, fetal growth trajectory was summarized by a change in an EFW SDS from second trimester to birth. These results are broadly consistent with our findings of an increase in mean AL and mean CR for groups with larger HC in late gestation, as well as similar relationships observed in the EFW and FL models, which likely reflect the close correlation that exists between these three fetal growth parameters.^32^

However, the fact that in our study, the associations between HC trajectory group membership and AL did not reach statistical significance suggests that by 20 years of age, the relationship between HC growth trajectory and AL has likely been diminished by the influence of environmental risk factors for myopia introduced during childhood, such as reduced time spent outdoors or increased time spent doing near work. In contrast, it seems that the proportional relationship between HC growth trajectory and CR that develops during fetal life remains stable into adolescence and adulthood. These observations are consistent with existing studies that indicate while axial elongation can continue into late adolescence, flattening of the cornea predominantly takes place during infancy as a largely passive component of emmetropization, the coordinated scaling process that occurs from birth to 12 to 18 months in order to achieve optimal refraction.^33^^–^^36^ Therefore, after this period, further compensatory changes in corneal shape cannot occur in the case of excessive axial elongation. In fact, several cross-sectional studies have found that while AL and CR are strongly correlated in emmetropes, a much weaker relationship exists between these two parameters in those with myopia and in whom, therefore, emmetropization has been disrupted.^34^^,^^37^

Investigations with larger study populations are required to establish whether the trend toward a higher prevalence of myopia in groups reflecting faster fetal HC growth in this analysis was merely a chance finding or does indeed reflect a true relationship between these two variables. Regardless, the lack of a statistically significant association between myopia and trajectory group membership in the HC model indicates a high likelihood that if any additional risk of myopia is indeed conferred by faster HC growth, it is modified considerably by more important environmental risk factors for myopia present during childhood and adolescence. In the case that a relationship does exist, several potential underlying mechanisms could be further explored. It can be noted that existing studies into the relationship between orbital volume and ocular growth have shown conflicting results and involved analyses of small study populations.^38^^–^^40^ Further research involving larger sample sizes is therefore warranted to understand whether the observed trends in our HC model may be linked to differences in the size of the orbital cavity and, correspondingly, the available space for ocular expansion in the axial direction. Alternatively, it is possible that the presence of an environmental exposure or genetic predisposition, or a combination of these factors, may render individuals more prone to both faster fetal skeletal growth and excessive axial elongation during childhood. Indeed, in our analysis, higher proportions of participants with at least one parent with myopia, and therefore a genetic predisposition toward myopia, were determined in groups representing faster fetal growth across all four trajectory models, most prominently in the AC model. It can be noted that among the many genes conferring susceptibility to myopia identified in genome-wide association studies, several are involved in extracellular matrix organization both within the eye and in the musculoskeletal system.^41^^–^^43^ Subtle variations in the expression of such genes could partially explain the patterns observed in our results.

The U-shaped trend seen in the FL model with respect to myopia may in part be attributable to a confounding relationship between HC and FL growth. As previously stated, these two parameters are both markers of skeletal growth and tend to be very closely correlated during early life.^32^ Indeed, while only 56.5% of the study population was allocated to the “big”, “accelerated”, or “large” HC group, participants from these groups represented 82.0% of participants in the “large” FL group. The relatively high prevalence of myopia found in the “large” FL group may therefore reflect the fact that these participants were more likely to be members of these three HC groups, which each had a relatively high prevalence of myopia. However, this does not account for the high prevalence of myopia also observed in the “small” and “medium” FL groups. Indeed, U-shaped relationships in disease epidemiology often represent the influence of different processes occurring at either end of a spectrum, resulting in the disruption of normal biological development and function at both ends.^44^^–^^46^ Therefore, it is likely that the increased prevalence of myopia in the “small” and “medium” groups reflects a different set of genetic or environmental factors that slowed the intrauterine FL growth of these participants and concurrently disrupted the coordination between AL and CR during ocular development. One potential such example could be hypertension in pregnancy: our finding that this condition was more prevalent in the “small” FL group compared with the other trajectory groups in our analysis is consistent with existing research that has demonstrated an association between pregnancy-induced hypertension, especially preeclampsia, and fetal growth restriction, including shorter FL.^47^^,^^48^

It can be noted that the association between fetal FL growth trajectory and the development of myopia is statistically significant, indicating that this relationship is not appreciably modified by processes affecting refractive error that take place in response to environmental stimuli present during school-age years. It is acknowledged, however, that the proposed mechanistic explanations are merely speculative. Since both fetal growth and the development of myopia involve a complex interplay of genetic and environmental influences, more detailed investigation is required to elaborate the specific pathophysiology underlying the observed trends and to quantify the relative extent to which different risk factors contribute toward refractive error in young adults, which was not formally assessed in this study.

The main strengths of our study are its prospective design and the unique longitudinal data set containing both serial fetal biometry measurements performed during gestation and measurements of ophthalmic outcomes in early adulthood, with a narrow age range for follow-up. Much of the existing research into early life influences on the development of myopia has been based on cross-sectional data and has focused on postnatal surrogate markers for fetal growth, including birth weight, length, and HC. Prior studies have found no consistent relationship between these neonatal biometric parameters and refractive error measured at less than 10 years of age, despite statistically significant correlations between these parameters and AL and CR.^11^^–^^14^ In contrast, modest but statistically significant associations between birth weight and refractive error measured in adulthood have been found in analyses from the 1958 British birth cohort^10^ and the Gutenberg Health Study,^15^ which both suggest that individuals with lower birth weight for gestational age have an increased risk of developing myopia. These results are supported by a recent UK study^16^ that used a Mendelian randomization study design to determine the causal influence of birth weight on refractive error in a population of 37- to 73-year-old UK Biobank participants. The effect in this study was estimated to be +0.28 D (95% confidence interval, 0.05–0.52, *P* = 0.02) per standard deviation increase in birth weight when adjusted for age and gender. The apparent discrepancy between the results from studies in adult and child populations may be accounted for by the fact that the typical age of onset for myopia is between 9 and 12 years.^49^ The possibility of study participants developing myopia subsequent to completion of follow-up therefore does not apply to the three latter studies or to our analysis, since follow-up in these studies was conducted after all participants had reached adulthood. The fact that the patterns observed in the EFW model with respect to myopia in our analysis do not appear to align with the associations between birth weight and refractive error measured in adulthood demonstrated in previous studies may reflect a difference between the characterization of fetal growth via either trajectory modeling or birth weight, the latter of which has been shown to be unreliable as a surrogate marker for fetal growth.^50^

Our study was primarily limited by its modest sample size, which resulted in some groups, particularly those representing either accelerating growth trajectories or consistently small or large fetal size, containing very small numbers of participants. Furthermore, none of the HC, AC, or FL models included groups representing participants whose growth rate decelerated throughout gestation relative to the rest of the study population, and so we were unable to fully assess the effects of intrauterine growth restriction with respect to these parameters. It is likely that the relatively small sample size resulted in a very small number of participants with decelerated growth. GBTM is known to have poor efficacy in detecting subgroups representing only a small proportion of a study population,^51^^,^^52^ and therefore it is probable that the participants with decelerated growth were not identified as a separate trajectory group in the analysis. Further investigation involving larger study populations will be needed to confirm the reliability and validity of our observed results.

The Gen2-20 year follow-up was the first time a comprehensive ophthalmic examination was conducted on the Gen2 of the Raine Study, and so our study is also limited by the lack of ophthalmic data from earlier ages. Serial ophthalmic assessments across different ages in future birth cohorts may strengthen the evidence for the associations identified in this study and provide a further understanding of how fetal development may influence the growth of the eye through childhood and adolescence. Moreover, despite the unique nature of our data set in containing a wide range of variables relevant to fetal growth during pregnancy and myopia in young adulthood, it should be noted that data related to the variables of parental myopia, total area of CUVAF, and education status were recorded for only just under 80% of the study population. Analyses within this study that include these variables may therefore be subject to bias associated with these missing data.

In addition, given the ethnic homogeneity of the study population, the results of our analysis are exclusively applicable to Caucasian populations. The risk of developing myopia is known to vary considerably with ethnicity: for example, a 2016 meta-analysis^53^ determined that the prevalence of myopia was 40% in 15-year-olds of either South Asian or Southeast Asian heritage living in Australia in contrast to a prevalence of just 17% among 15-year-olds of Caucasian descent. Similarly, trajectories of fetal biometry measures are significantly influenced by ethnicity.^54^ Inclusion of larger proportions of individuals of non-Caucasian heritage in cohorts for future investigations will be necessary to establish whether similar associations to those observed in this study exist in other ethnic groups.

In conclusion, our results show that specific aspects of intrauterine skeletal growth correlate with the risk of myopia and alterations in ocular biometry. We hypothesize that these observations reflect processes that concurrently influence skeletal growth in utero as well as the coordination between axial elongation and corneal flattening during eye development, with effects that persist into adulthood, albeit with modification by environmental factors present during childhood and adolescence. Our findings provide a basis for further investigating both genetic and intrauterine environmental factors underlying the observed associations. This research may also lead to methods for identifying infants at risk of developing myopia as early as 38 weeks’ gestation and for implementing proven preventative strategies for these individuals, for example, increased time spent outdoors.

## Supplementary Material

Supplement 1

Supplement 2

Supplement 3

Supplement 4

Supplement 5

Supplement 6

Supplement 7
