# Supplementary material for: Associations Between Fetal Growth Trajectories and the Development of Myopia by 20 Years of Age
Source: Invest Ophthalmol Vis Sci. 2020 Dec 23;61(14):26. doi: 10.1167/iovs.61.14.26 (PMC7774062; doi:10.1167/iovs.61.14.26)
Supplement: Supplement 4 [file iovs-61-14-26_s004.pdf]

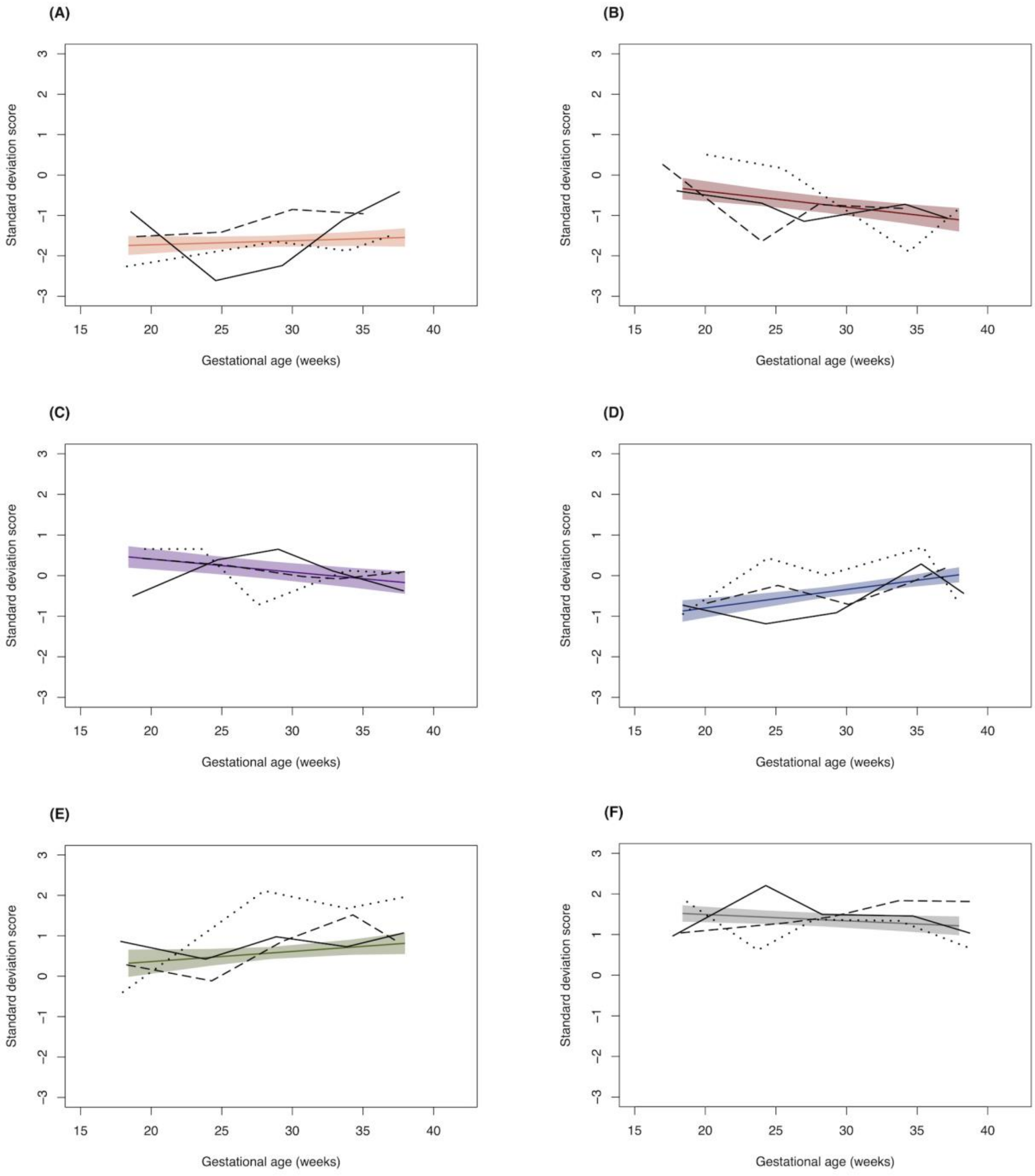

<u>Supplementary Figure S4</u>: Estimated fetal weight trajectory model with individual profiles. (A) “Small” trajectory group; (B) “medium-small” trajectory group; (C) “big-medium” trajectory group; (D) “medium-big” trajectory group; (E) “big-large” trajectory group; and (F) “large” trajectory group from the estimated fetal weight model. Each panel presents the trajectories of three randomly selected individuals (solid, dashed and dotted black lines) from the respective trajectory group, along with the estimated mean trajectory for the group with a 95% confidence interval.
