# Supplementary material for: Associations Between Fetal Growth Trajectories and the Development of Myopia by 20 Years of Age
Source: Invest Ophthalmol Vis Sci. 2020 Dec 23;61(14):26. doi: 10.1167/iovs.61.14.26 (PMC7774062; doi:10.1167/iovs.61.14.26)
Supplement: Supplement 5 [file iovs-61-14-26_s005.docx]

Supplementary Table S1: Maternal and gestational characteristics of participants in the trajectory groups of the four models.

|  | Trajectory (Head Circumference Model) | | | | |  | Trajectory (Femur Length Model) | | | | | |  |
| --- | --- | --- | --- | --- | --- | --- | --- | --- | --- | --- | --- | --- | --- |
|  | Small | Medium | Big | Accelerated | Large | *p*-value | Small | Medium | Big | Accelerated | Large | | *p*-value |
| n (%) | 29 (6.6 %) | 162 (36.9%) | 172 (39.2%) | 46 (10.5%) | 30 (6.8%) |  | 34 (6.8%) | 159 (32.1%) | 197 (39.7%) | 48 (9.7%) | 58 (11.7%) | |  |
|  | Pregnancy characteristics | | | | |  | Pregnancy characteristics | | | | |  | |
| Maternal age (years) | 29.65 ± 5.87 | 29.49 ± 5.49 | 29.62 ± 5.81 | 29.05 ± 5.46 | 28.67 ± 5.77 | 0.91 | 30.91 ± 5.61 | 29.4 ± 6.06 | 29.7 ± 5.50 | 28.81 ± 5.45 | 28.81 ± 5.88 | | 0.42 |
| Maternal height (m) | 1.63 ± 0.08 | 1.64 ± 0.07 | 1.65 ± 0.06 | 1.64 ± 0.06 | 1.63 ± 0.06 | 0.53 | 1.63 ± 0.06 | 1.65 ± 0.07 | 1.64 ± 0.06 | 1.66 ± 0.06 | 1.64 ± 0.05 | | 0.15 |
| Maternal weight (kg) | 59.10 ± 11.90 | 59.90 ± 10.04 | 59.78 ± 10.42 | 62.11 ± 14.21 | 61.70 ± 11.19 | 0.62 | 57.62 ± 12.21 | 60.91 ± 10.62 | 58.94 ± 11.23 | 62.50 ± 10.78 | 61.69 ± 10.81 | | 0.09 |
| Rate of gestational weight gain (kg/week) | 0.48 ± 0.21 | 0.49 ± 0.19 | 0.53 ± 0.20 | 0.53 ± 0.20 | 0.45 ± 0.24 | 0.19 | 0.46 ± 0.19 | 0.50 ± 0.20 | 0.50 ± 0.17 | 0.50 ± 0.16 | 0.58 ± 0.26 | | 0.03^*^ |
| Parity |  |  |  |  |  |  |  |  |  |  |  | |  |
| 0 | 20 (69.0%) | 79 (48.8%) | 93 (54.1%) | 24 (52.2%) | 18 (60.0%) | 0.30 | 18 (52.9%) | 85 (53.5%) | 99 (50.3%) | 24 (50.0%) | 35 (60.3%) | | 0.73 |
| 1+ | 9 (31.0%) | 83 (51.2%) | 79 (45.9%) | 22 (47.8%) | 12 (40.0%) |  | 16 (47.1%) | 74 (46.5%) | 98 (49.7%) | 24 (50.0%) | 23 (39.7%) | |  |
| Sex |  |  |  |  |  |  |  |  |  |  |  | |  |
| Male | 15 (51.7%) | 76 (46.9%) | 90 (52.3%) | 26 (56.5%) | 16 (53.3%) | 0.77 | 21 (61.8%) | 78 (49.1%) | 102 (51.8%) | 19 (39.6%) | 29 (50.0%) | | 0.37 |
| Female | 14 (48.3%) | 86 (53.1%) | 82 (47.7%) | 20 (43.5%) | 14 (46.7%) |  | 13 (38.2%) | 81 (50.9%) | 95 (48.2%) | 29 (60.4%) | 29 (50.0%) | |  |
| Hypertension in pregnancy | |  |  |  |  |  |  |  |  |  |  | |  |
| Yes | 7 (24.1%) | 46 (28.4%) | 32 (18.6%) | 14 (30.4%) | 10 (33.3%) | 0.15 | 14 (41.2%) | 33 (20.8%) | 45 (22.8%) | 14 (29.2%) | 15 (25.9%) | | 0.15 |
| No | 22 (75.9%) | 116 (71.6%) | 140 (81.4%) | 32 (69.6%) | 20 (66.7%) |  | 20 (58.8%) | 126 (79.2%) | 152 (77.2%) | 34 (70.8%) | 43 (74.1%) | |  |
| Pre-existing or gestational diabetes | |  |  |  |  |  |  |  |  |  |  | |  |
| Yes | 0 (0.0%) | 7 (4.3%) | 7 (4.1%) | 0 (0.0%) | 3 (10.0%) | 0.09 | 2 (5.9%) | 2 (1.3%) | 11 (5.6%) | 2 (4.2%) | 3 (5.2%) | | 0.21 |
| No | 29 (100.0%) | 155 (95.7%) | 165 (95.9%) | 46 (100.0%) | 27 (90.0%) |  | 32 (94.1%) | 157 (98.7%) | 186 (94.4%) | 46 (95.8%) | 55 (94.8%) | |  |
| Gestational anemia | |  |  |  |  |  |  |  |  |  |  | |  |
| Yes | 8 (27.6%) | 38 (23.5%) | 47 (27.3%) | 12 (26.1%) | 13 (43.3%) | 0.32 | 5 (14.7%) | 48 (30.2%) | 53 (26.9%) | 14 (29.2%) | 11 (19.0%) | | 0.20 |
| No | 21 (72.4%) | 123 (75.9%) | 125 (72.7%) | 34 (73.9%) | 17 (56.7%) |  | 29 (85.3%) | 110 (69.2%) | 144 (73.1%) | 34 (70.8%) | 47 (81.0%) | |  |
| Data missing | 0 (0.0%) | 1 (0.6%) | 0 (0.0%) | 0 (0.0%) | 0 (0.0%) |  | 0 (0.0%) | 1 (0.6%) | 0 (0.0%) | 0 (0.0%) | 0 (0.0%) | |  |
| Smoking during pregnancy | |  |  |  |  |  |  |  |  |  |  | |  |
| Yes | 10 (34.5%) | 40 (24.7%) | 38 (22.1%) | 6 (13.0%) | 2 (6.7%) | 0.03^*^ | 14 (41.2%) | 39 (24.5%) | 38 (19.3%) | 9 (18.8%) | 7 (12.1%) | | 0.02^*^ |
| No | 19 (65.5%) | 122 (75.3%) | 134 (77.9%) | 40 (87.0%) | 28 (93.3%) |  | 20 (58.8%) | 120 (75.5%) | 159 (80.7%) | 39 (81.2%) | 51 (87.9%) | |  |
| Abnormal Doppler flow | |  |  |  |  |  |  |  |  |  |  | |  |
| Yes | 3 (10.3%) | 21 (13.0%) | 11 (6.4%) | 1 (2.2%) | 3 (10.0%) | 0.09 | 4 (11.8%) | 9 (5.7%) | 20 (10.2%) | 6 (12.5%) | 4 (6.9%) | | 0.41 |
| No | 26 (89.7%) | 141 (87.0%) | 161 (93.6%) | 45 (97.8%) | 27 (90.0%) |  | 30 (88.2%) | 150 (94.3%) | 177 (89.8%) | 42 (87.5%) | 54 (93.1%) | |  |

|  | Trajectory (Abdominal Circumference Model) | | | | | | | | | |  | Trajectory (Estimated Fetal Weight Model) | | | | | | | | |  |
| --- | --- | --- | --- | --- | --- | --- | --- | --- | --- | --- | --- | --- | --- | --- | --- | --- | --- | --- | --- | --- | --- |
|  | Small | | | Medium | | | Accelerated | | Large | | *p*-value | Small | | Medium-Small | | Big-Medium | Medium-Big | Big-Large | Large | | *p*-value |
| n (%) | 98 (20.0%) | | | 229 (46.7%) | | | 57 (11.6%) | | 106 (21.6%) | |  | 34 (7.9%) | | 52 (12.1%) | | 114 (26.6%) | 93 (21.7%) | 91 (21.2%) | 45 (10.5%) | |  |
|  | Pregnancy characteristics | | | | | | | | | | |  | Pregnancy characteristics | | | | | | |  | |
| Maternal age (years) | 29.61 ± 5.33 | | | 29.21 ± 5.81 | | | 30.67 ± 5.05 | | 29 ± 5.95 | | 0.29 | 29.64 ± 5.55 | | 29.02 ± 4.97 | 28.95 ± 6.37 | | 29.31 ± 5.21 | 29.88 ± 5.05 | 29.66 ± 6.59 | | 0.88 |
| Maternal height (m) | 1.65 ± 0.08 | | | 1.64 ± 0.06 | | | 1.65 ± 0.06 | | 1.64 ± 0.06 | | 0.66 | 1.63 ± 0.08 | | 1.64 ± 0.06 | 1.64 ± 0.06 | | 1.65 ± 0.06 | 1.64 ± 0.06 | 1.64 ± 0.06 | | 0.63 |
| Maternal weight (kg) | 60.09 ± 11.63 | | | 59.14 ± 10.47 | | | 61.30 ± 11.93 | | 62.00 ± 11.07 | | 0.14 | 59.41 ± 12.31 | | 57.44 ± 7.86 | 59.08 ± 11.75 | | 62.32 ± 12.19 | 60.70 ± 8.98 | 61.31 ± 11.07 | | 0.11 |
| Rate of gestational weight gain (kg/week) | 0.49 ± 0.20 | | | 0.51 ± 0.18 | | | 0.53 ± 0.20 | | 0.51 ± 0.22 | | 0.55 | 0.46 ± 0.20 | | 0.50 ± 0.18 | 0.48 ± 0.18 | | 0.51 ± 0.19 | 0.55 ± 0.19 | 0.53 ± 0.26 | | 0.12 |
| Parity |  | | |  | | |  | |  | |  |  | |  |  | |  |  |  | |  |
| 0 | 53 (54.1%) | | | 117 (51.1%) | | | 32 (56.1%) | | 52 (49.1%) | | 0.80 | 18 (52.9%) | | 25 (48.1%) | 56 (49.1%) | | 57 (61.3%) | 47 (51.6%) | 22 (48.9%) | | 0.53 |
| 1+ | 45 (45.9%) | | | 112 (48.9%) | | | 25 (43.9%) | | 54 (50.9%) | |  | 16 (47.1%) | | 27 (51.9%) | 58 (50.9%) | | 36 (38.7%) | 44 (48.4%) | 23 (51.1%) | |  |
| Sex |  | | |  | | |  | |  | |  |  | |  |  | |  |  |  | |  |
| Male | 52 (53.1%) | | | 117 (51.1%) | | | 22 (38.6%) | | 54 (50.9%) | | 0.32 | 16 (47.1%) | | 30 (57.7%) | 57 (50.0%) | | 47 (50.5%) | 48 (52.7%) | 21 (46.7%) | | 0.90 |
| Female | 46 (46.9%) | | | 112 (48.9%) | | | 35 (61.4%) | | 52 (49.1%) | |  | 18 (52.9%) | | 22 (42.3%) | 57 (50.0%) | | 46 (49.4%) | 43 (47.3%) | 24 (53.3%) | |  |
| Hypertension in pregnancy | | |  | | |  | |  | | |  |  | |  |  | |  |  |  | |  |
| Yes | 28 (28.6%) | | | 48 (21.0%) | | | 19 (33.3%) | | 26 (24.5%) | | 0.20 | 9 (26.5%) | | 11 (21.2%) | 25 (21.9%) | | 28 (30.1%) | 19 (20.9%) | 15 (33.3%) | | 0.45 |
| No | 70 (71.4%) | | | 181 (79.0%) | | | 38 (66.7%) | | 80 (75.5%) | |  | 25 (73.5%) | | 41 (78.8%) | 89 (78.1%) | | 65 (69.9%) | 72 (79.1%) | 30 (66.7%) | |  |
| Pre-existing or gestational diabetes | | |  | | |  | |  | | |  |  | |  |  | |  |  |  | |  |
| Yes | 4 (4.1%) | | | 3 (1.3%) | | | 4 (7.0%) | | 8 (7.5%) | | 0.02^*^ | 1 (2.9%) | | 2 (3.8%) | 4 (3.5%) | | 1 (1.1%) | 4 (4.4%) | 4 (8.9%) | | 0.40 |
| No | 94 (95.9%) | | | 226 (98.7%) | | | 53 (93.0%) | | 98 (92.5%) | |  | 33 (97.1%) | | 50 (96.2%) | 110 (96.5%) | | 92 (98.9%) | 87 (95.6%) | 41 (91.1%) | |  |
| Gestational anemia | | |  | | |  | |  | | |  |  | |  |  | |  |  |  | |  |
| Yes | 21 (21.4%) | | | 58 (25.3%) | | | 19 (33.3%) | | 32 (30.2%) | | 0.29 | 8 (23.5%) | | 8 (15.4%) | 31 (27.2%) | | 25 (26.9%) | 27 (29.7%) | 15 (33.3%) | | 0.37 |
| No | 77 (78.6%) | | | 171 (74.7%) | | | 37 (64.9%) | | 74 (69.8%) | |  | 26 (76.5%) | | 44 (84.6%) | 83 (72.8%) | | 67 (72.0%) | 64 (70.3%) | 30 (66.7%) | |  |
| Data missing | 0 (0.0%) | | | 0 (0.0%) | | | 1 (1.8%) | | 0 (0.0%) | |  | 0 (0.0%) | | 0 (0.0%) | 0 (0.0%) | | 1 (1.1%) | 0 (0.0%) | 0 (0.0%) | |  |
| Smoking during pregnancy | | |  | | |  | |  | | |  |  | |  |  | |  |  |  | |  |
| Yes | 24 (24.5%) | | | 57 (24.9%) | | | 5 (8.8%) | | 19 (17.9%) | | 0.02^*^ | 12 (35.3%) | | 13 (25.0%) | 30 (26.3%) | | 22 (23.7%) | 14 (15.4%) | 4 (8.9%) | | 0.03^*^ |
| No | 74 (75.5%) | | | 172 (75.1%) | | | 52 (91.2%) | | 87 (82.1%) | |  | 22 (64.7%) | | 39 (75.0%) | 84 (73.7%) | | 71 (76.3%) | 77 (84.6%) | 41 (91.1%) | |  |
| Abnormal Doppler flow | |  | | |  | | | | |  |  |  | |  |  | |  |  |  | |  |
| Yes | 14 (14.3%) | | | 14 (6.1%) | | | 4 (7.0%) | | 10 (9.4%) | | 0.13 | 6 (17.6%) | | 7 (13.5%) | 14 (12.3%) | | 4 (4.3%) | 3 (3.3%) | 5 (11.1%) | | 0.02^*^ |
| No | 84 (85.7%) | | | 215 (93.9%) | | | 53 (93.0%) | | 96 (90.6%) | |  | 28 (82.4%) | | 45 (86.5%) | 100 (87.7%) | | 89 (95.7%) | 88 (96.7%) | 40 (88.9%) | |  |

Data are summarized by number and percentage of the trajectory group for categorical variables or by mean and standard deviation for continuous variables.

*p*-values have been calculated using binary logistic regression for categorical data and one-way analysis of variance for continuous data.

**^*^**Significant at *p* < 0.05.
