# Supplementary material for: Associations Between Fetal Growth Trajectories and the Development of Myopia by 20 Years of Age
Source: Invest Ophthalmol Vis Sci. 2020 Dec 23;61(14):26. doi: 10.1167/iovs.61.14.26 (PMC7774062; doi:10.1167/iovs.61.14.26)
Supplement: Supplement 6 [file iovs-61-14-26_s006.docx]

Supplementary Table S2: Distributions of known risk factors for myopia across the trajectory groups of the four models.

|  | Trajectory (Head Circumference Model) | | | | | | | | | | | |  |
| --- | --- | --- | --- | --- | --- | --- | --- | --- | --- | --- | --- | --- | --- |
|  | Small | | Medium | | | Big | | Accelerated | | | Large | | *p*-value |
| n (%) | 29 (6.6 %) | | 162 (36.9%) | | | 172 (39.2%) | | 46 (10.5%) | | | 30 (6.8%) | |  |
| Gestational age at birth (weeks) | 39.9 ± 1.52 | | 39.73 ± 1.35 | | | 39.77 ± 1.43 | | 40.15 ± 1.88 | | | 39.06 ± 1.6 | | 0.04* |
| Birth weight score^†^ | -0.94 ± 0.89 | | -0.41 ± 0.9 | | | 0.35 ± 0.9 | | 0.2 ± 0.86 | | | 0.78 ± 0.85 | | <0.01* |
| Total area of CUVAF (mm^2^) | 42.21 (29.84, 58.47) | | 39.02 (15.60, 66.80) | | | 49.11 (20.73, 78.15) | | 51.73 (26.17, 85.10) | | | 46.78 (27.73, 67.29) | | 0.19 |
| Parental myopia |  | |  | | |  | |  | | |  | |  |
| Neither | 19 (65.52%) | | 111 (68.52%) | | | 98 (56.98%) | | 29 (63.04%) | | | 15 (50%) | | 0.67 |
| One | 4 (13.79%) | | 21 (12.96%) | | | 25 (14.53%) | | 9 (19.57%) | | | 6 (20%) | |  |
| Both | 1 (3.45%) | | 7 (4.32%) | | | 10 (5.81%) | | 0 (0%) | | | 1 (3.33%) | |  |
| Missing | 5 (17.24%) | | 23 (14.2%) | | | 39 (22.67%) | | 8 (17.39%) | | | 8 (26.67%) | |  |
| Education level |  | |  | | |  | |  | | |  | |  |
| Completed Year 12 | 20 (68.97%) | | 101 (62.35%) | | | 106 (61.63%) | | 34 (73.91%) | | | 19 (63.33%) | | 0.13 |
| Did not complete | 4 (13.79%) | | 38 (23.46%) | | | 27 (15.7%) | | 4 (8.7%) | | | 3 (10%) | |  |
| Missing | 5 (17.24%) | | 23 (14.2%) | | | 39 (22.67%) | | 8 (17.39%) | | | 8 (26.67%) | |  |
|  | Trajectory (Abdominal Circumference Model) | | | | | | | | | | | |  |
|  | Small | | | Medium | | | Accelerated | | | Large | | | *p*-value |
| n (%) | 98 (20.0%) | | | 229 (46.7%) | | | 57 (11.6%) | | | 106 (21.6%) | | |  |
| Gestational age at birth (weeks) | 39.59 ± 1.93 | | | 39.72 ± 1.44 | | | 39.9 ± 1.29 | | | 39.46 ± 1.41 | | | 0.27 |
| Birth weight score^†^ | -0.85 ± 0.85 | | | -0.18 ± 0.74 | | | 0.71 ± 0.79 | | | 0.78 ± 0.92 | | | <0.01* |
| Total area of CUVAF (mm^2^) | 40.71 (20.19, 69.02) | | | 45.54 (22.46, 75.77) | | | 46.62 (26.27, 80.41) | | | 47.34 (26.85, 66.36) | | | 0.59 |
| Parental myopia |  | | |  | | |  | | |  | | |  |
| Neither | 66 (67.35%) | | | 150 (65.5%) | | | 34 (59.65%) | | | 50 (47.17%) | | | <0.01* |
| One | 15 (15.31%) | | | 27 (11.79%) | | | 7 (12.28%) | | | 28 (26.42%) | | |  |
| Both | 3 (3.06%) | | | 9 (3.93%) | | | 4 (7.02%) | | | 5 (4.72%) | | |  |
| Missing | 14 (14.29%) | | | 43 (18.78%) | | | 12 (21.05%) | | | 23 (21.7%) | | |  |
| Education level |  | | |  | | |  | | |  | | |  |
| Completed Year 12 | 65 (66.33%) | | | 148 (64.63%) | | | 39 (68.42%) | | | 66 (62.26%) | | | 0.62 |
| Did not complete | 19 (19.39%) | | | 38 (16.59%) | | | 6 (10.53%) | | | 17 (16.04%) | | |  |
| Missing | 14 (14.29%) | | | 43 (18.78%) | | | 12 (21.05%) | | | 23 (21.7%) | | |  |
|  | Trajectory (Femur Length Model) | | | | | | | | | | | |  |
|  | Small | | Medium | | | Big | | Accelerated | | | Large | | *p*-value |
| n (%) | 34 (6.8%) | | 159 (32.1%) | | | 197 (39.7%) | | 48 (9.7%) | | | 58 (11.7%) | |  |
| Gestational age at birth (weeks) | 39.35 ± 1.72 | | 39.78 ± 1.58 | | | 39.61 ± 1.45 | | 39.79 ± 1.84 | | | 39.2 ± 1.52 | | 0.12 |
| Birth weight score^†^ | -0.89 ± 1.03 | | -0.2 ± 0.86 | | | 0.16 ± 0.98 | | -0.34 ± 0.81 | | | 0.83 ± 0.86 | | <0.01* |
| Total area of CUVAF (mm^2^) | 47.22 (30.20, 82.23) | | 42.89 (19.80, 72.07) | | | 47.84 (21.57, 74.28) | | 38.32 (13.72, 66.56) | | | 43.33 (27.59, 65.42) | | 0.40 |
| Parental myopia |  | |  | | |  | |  | | |  | |  |
| Neither | 20 (58.82%) | | 105 (66.04%) | | | 119 (60.41%) | | 29 (60.42%) | | | 29 (50%) | | 0.20 |
| One | 4 (11.76%) | | 25 (15.72%) | | | 30 (15.23%) | | 9 (18.75%) | | | 12 (20.69%) | |  |
| Both | 2 (5.88%) | | 5 (3.14%) | | | 6 (3.05%) | | 2 (4.17%) | | | 7 (12.07%) | |  |
| Missing | 8 (23.53%) | | 24 (15.09%) | | | 42 (21.32%) | | 8 (16.67%) | | | 10 (17.24%) | |  |
| Education level |  | |  | | |  | |  | | |  | |  |
| Completed Year 12 | 20 (58.82%) | | 107 (67.3%) | | | 124 (62.94%) | | 32 (66.67%) | | | 39 (67.24%) | | 0.99 |
| Did not complete | 6 (17.65%) | | 28 (17.61%) | | | 31 (15.74%) | | 8 (16.67%) | | | 9 (15.52%) | |  |
| Missing | 8 (23.53%) | | 24 (15.09%) | | | 42 (21.32%) | | 8 (16.67%) | | | 10 (17.24%) | |  |
|  | Trajectory (Estimated Fetal Weight Model) | | | | | | | | | | | |  |
|  | Small | Medium-Small | | | Big-Medium | | Medium-Big | | Big-Large | | | Large | *p*-value |
| n (%) | 34 (7.9%) | 52 (12.1%) | | | 114 (26.6%) | | 93 (21.7%) | | 91 (21.2%) | | | 45 (10.5%) |  |
| Gestational age at birth (weeks) | 39.66 ± 1.93 | 39.85 ± 1.43 | | | 39.61 ± 1.45 | | 40.05 ± 1.29 | | 39.87 ± 1.31 | | | 39.4 ± 1.62 | 0.13 |
| Birth weight score^†^ | -1.21 ± 0.7 | -0.91 ± 0.69 | | | -0.13 ± 0.75 | | -0.07 ± 0.72 | | 0.76 ± 0.73 | | | 0.93 ± 0.96 | <0.01* |
| Total area of CUVAF (mm^2^) | 41.70 (23.58, 71.79) | 44.50 (21.95, 72.47) | | | 39.44 (17.56, 66.41) | | 45.97 (17.97, 71.80) | | 51.92 (24.47, 73.98) | | | 46.48 (22.70, 66.24) | 0.71 |
| Parental myopia |  |  | | |  | |  | |  | | |  |  |
| Neither | 23 (67.65%) | 36 (69.23%) | | | 69 (60.53%) | | 65 (69.89%) | | 53 (58.24%) | | | 21 (46.67%) | 0.08 |
| One | 3 (8.82%) | 6 (11.54%) | | | 14 (12.28%) | | 11 (11.83%) | | 17 (18.68%) | | | 10 (22.22%) |  |
| Both | 1 (2.94%) | 2 (3.85%) | | | 5 (4.39%) | | 2 (2.15%) | | 4 (4.4%) | | | 4 (8.89%) |  |
| Missing | 7 (20.59%) | 8 (15.38%) | | | 26 (22.81%) | | 15 (16.13%) | | 17 (18.68%) | | | 10 (22.22%) |  |
| Education level |  |  | | |  | |  | |  | | |  |  |
| Completed Year 12 | 23 (67.65%) | 36 (69.23%) | | | 60 (52.63%) | | 60 (64.52%) | | 63 (69.23%) | | | 31 (68.89%) | 0.06 |
| Did not complete | 4 (11.76%) | 8 (15.38%) | | | 28 (24.56%) | | 18 (19.35%) | | 11 (12.09%) | | | 4 (8.89%) |  |
| Missing | 7 (20.59%) | 8 (15.38%) | | | 26 (22.81%) | | 15 (16.13%) | | 17 (18.68%) | | | 10 (22.22%) |  |

Data are summarized by number and percentage of the trajectory group for categorical variables or by mean and standard deviation for continuous variables (median and IQR if non-parametric data).

*p*-values have been calculated using binary logistic regression for categorical data and one-way analysis of variance for continuous data (Kruskal-Wallis test if non-parametric data).

**^*^**Significant at *p* < 0.05.

^†^Birth weight adjusted for gestational age at birth. Calculated by constructing a linear regression model for birth weight with respect to gestational age and taking the standardized residuals as the adjusted birth weights.
