# Supplementary material for: Associations Between Fetal Growth Trajectories and the Development of Myopia by 20 Years of Age
Source: Invest Ophthalmol Vis Sci. 2020 Dec 23;61(14):26. doi: 10.1167/iovs.61.14.26 (PMC7774062; doi:10.1167/iovs.61.14.26)
Supplement: Supplement 7 [file iovs-61-14-26_s007.docx]

Supplementary Table S3: Ophthalmic characteristics of participants in the trajectory groups of the four models.

|  | Trajectory (Head Circumference Model) | | | | | | | | | | | |  |
| --- | --- | --- | --- | --- | --- | --- | --- | --- | --- | --- | --- | --- | --- |
|  | Small | | Medium | | | Big | | Accelerated | | | Large | | *p*-value |
| n (%) | 29 (6.6 %) | | 162 (36.9%) | | | 172 (39.2%) | | 46 (10.5%) | | | 30 (6.8%) | |  |
| Axial length (mm) | 23.37 (23.05 to 23.69) | | 23.44 (23.30 to 23.57) | | | 23.67 (23.54 to 23.80) | | 23.56 (23.30 to 23.81) | | | 23.64 (23.32 to 23.96) | | 0.12 |
| Corneal radius of curvature (mm) | 7.69 (7.60 to 7.78) | | 7.70 (7.66 to 7.74) | | | 7.78 (7.74 to 7.82) | | 7.79 (7.71 to 7.86) | | | 7.79 (7.70 to 7.89) | | 0.03^*^ |
| Myopia |  | |  | | |  | |  | | |  | |  |
| Yes | 4 (13.8%) | | 32 (19.8%) | | | 33 (19.2%) | | 10 (21.7%) | | | 7 (23.3%) | | 0.90 |
| No | 25 (86.2%) | | 130 (80.2%) | | | 139 (80.8%) | | 36 (78.3%) | | | 23 (76.7%) | |  |
|  | Trajectory (Abdominal Circumference Model) | | | | | | | | | | | |  |
|  | Small | | | Medium | | | Accelerated | | | Large | | | *p*-value |
| n (%) | 98 (20.0%) | | | 229 (46.7%) | | | 57 (11.6%) | | | 106 (21.6%) | | |  |
| Axial length (mm) | 23.54 (23.37 to 23.72) | | | 23.61 (23.49 to 23.73) | | | 23.49 (23.26 to 23.72) | | | 23.55 (23.38 to 23.72) | | | 0.79 |
| Corneal radius of curvature (mm) | 7.70 (7.65 to 7.75) | | | 7.75 (7.72 to 7.79) | | | 7.79 (7.72 to 7.86) | | | 7.76 (7.72 to 7.81) | | | 0.13 |
| Myopia |  | | |  | | |  | | |  | | |  |
| Yes | 21 (21.4%) | | | 45 (19.7%) | | | 11 (19.3%) | | | 20 (18.9%) | | | 0.97 |
| No | 77 (78.6%) | | | 184 (80.3%) | | | 46 (80.7%) | | | 86 (81.1%) | | |  |
|  | Trajectory (Femur Length Model) | | | | | | | | | | | |  |
|  | Small | | Medium | | | Big | | Accelerated | | | Large | | *p*-value |
| n (%) | 34 (6.8%) | | 159 (32.1%) | | | 197 (39.7%) | | 48 (9.7%) | | | 58 (11.7%) | |  |
| Axial length (mm) | 23.39 (23.09 to 23.69) | | 23.59 (23.45 to 23.72) | | | 23.53 (23.41 to 23.65) | | 23.5 (23.25 to 23.75) | | | 23.84 (23.61 to 24.07) | | 0.11 |
| Corneal radius of curvature (mm) | 7.63 (7.55 to 7.72) | | 7.73 (7.69 to 7.77) | | | 7.77 (7.73 to 7.80) | | 7.76 (7.69 to 7.84) | | | 7.79 (7.72 to 7.85) | | 0.04^*^ |
| Myopia |  | |  | | |  | |  | | |  | |  |
| Yes | 9 (26.5%) | | 36 (22.6%) | | | 27 (13.7%) | | 8 (16.7%) | | | 17 (29.3%) | | 0.04^*^ |
| No | 25 (73.5%) | | 123 (77.4%) | | | 170 (86.3%) | | 40 (83.3%) | | | 41 (70.7%) | |  |
|  | Trajectory (Estimated Fetal Weight Model) | | | | | | | | | | | |  |
|  | Small | Medium-Small | | | Big-Medium | | Medium-Big | | Big-Large | | | Large | *p*-value |
| n (%) | 34 (7.9%) | 52 (12.1%) | | | 114 (26.6%) | | 93 (21.7%) | | 91 (21.2%) | | | 45 (10.5%) |  |
| Axial length (mm) | 23.38 (23.08 to 23.68) | 23.44 (23.20 to 23.68) | | | 23.52 (23.36 to 23.68) | | 23.47 (23.29 to 23.65) | | 23.71 (23.53 to 23.9) | | | 23.62 (23.37 to 23.88) | 0.28 |
| Corneal radius of curvature (mm) | 7.67 (7.59 to 7.76) | 7.69 (7.62 to 7.76) | | | 7.72 (7.67 to 7.76) | | 7.74 (7.69 to 7.79) | | 7.82 (7.77 to 7.88) | | | 7.76 (7.68 to 7.83) | 0.01^*^ |
| Myopia |  |  | | |  | |  | |  | | |  |  |
| Yes | 4 (11.8%) | 10 (19.2%) | | | 22 (19.3%) | | 21 (22.6%) | | 19 (20.9%) | | | 7 (15.6%) | 0.76 |
| No | 30 (88.2%) | 42 (80.8%) | | | 92 (80.7%) | | 72 (77.4%) | | 72 (79.1%) | | | 38 (84.4%) |  |

Data are summarized by number and percentage of the trajectory group for categorical variables or by mean and 95% confidence interval, as calculated in a one-way analysis of variance, for continuous variables.

*p*-values have been calculated using binary logistic regression for categorical data and one-way analysis of variance for continuous data.

**^*^**Significant at *p* < 0.05.
